# Possible repurposing of seasonal influenza vaccine for prevention of Zika virus infection

**DOI:** 10.12688/f1000research.8102.2

**Published:** 2016-03-23

**Authors:** Veljko Veljkovic, Slobodan Paessler

**Affiliations:** 1 Biomed Protection, Galveston, TX, USA; 2 Department of Pathology, Galveston National Laboratory, University of Texas Medical Branch, Galveston, TX, USA; 3 Galveston National Laboratory, Institute for Human Infectious and Immunity, Galveston, TX, USA

**Keywords:** Zika virus, vaccine, influenza virus, vaccine repurposing, in silico analysis, hemagglutinin, envelope glycoprotein E, informational spectrum method

## Abstract

The *in silico* analysis shows that the envelope glycoproteins E of Zika viruses (ZIKV) isolated in Asia, Africa and South and Central America encode highly conserved information determining their interacting profile and immunological properties. Previously it was shown that the same information is encoded in the primary structure of the hemagglutinin subunit 1 (HA1) from pdmH1N1 influenza A virus.  This similarity suggests possible repurposing of the seasonal influenza vaccine containing pdmH1N1 component for prevention of the ZIKV infection.

## Introduction

The recent pandemic of the pdmH1N1 influenza virus and epidemic of the Ebola virus in West Africa showed a lack of preparedness to adequately respond to emerging infectious diseases with potential catastrophic consequences. One of the main obstacles to a fast and efficient response to emerging new infectious disease is insufficient knowledge of the biological, immunological and pathogenic properties of new pathogens and a lack of an appropriate experimental system for testing drugs and vaccines. The new emergence of the ZIKV showed that despite significant progress in molecular biology, biochemistry, immunology, medicine and pharmacology which allows better understanding, prevention and therapy of infectious diseases, the world once again is not prepared for early and decisive action which would prevent hundreds of unnecessary cases of ZIKV infections and potential congenital abnormalities in newborns caused by this virus.

Previously, at the beginning of the HIV epidemic ^1^, and recently during the swine flu pandemic ^2^ and Ebola epidemic in West Africa ^3^, we demonstrated that the bioinformatics tool which is based on the informational spectrum method (ISM) ^4^ can give some useful information about the host-pathogen interaction and help in selection of drug and vaccine candidates. An essential advantage of ISM over other bioinformatics approaches is in the use of DNA and protein sequences as the only input information which allows analysis of new pathogens. Because data about the sequencing of new pathogens usually are available at the beginning of the outbreaks, the ISM analysis can start immediately and provide some information which could accelerate development of vaccines and drugs.

The ZIKV, native to parts of Africa and Asia, has for the first time been introduced into the Americas. The ZIKV epidemic in Brazil currently is estimated at 440000–1300000 cases, and in February 2016 it has spread to other Latin-American countries, the USA and Europe ( http://www.thelancet.com/pdfs/journals/lancet/PIIS0140-6736(16)00014-3.pdf), threatening to become a pandemic. Recently, the World Health Organization (WHO) declared an international public health emergency ( http://www.who.int/mediacentre/news/statements/2016/emergency-committee-zika-microcephaly/en/). There is no vaccine against the virus or any antiviral treatment.

Here we analyzed ZIKV E proteins using ISM. Results of this *in silico* analysis revealed that these viral proteins encode the highly conserved information which determines their interacting profile and immunological properties. Previously, we reported that the human interacting profile of HA1 from pdmH1N1 influenza viruses is characterized by the same information ^2^. This result suggests possible repurposing of the seasonal influenza vaccine containing pdmH1N1 for prevention of ZIKV infection.

## Material and methods

### Sequences

All sequences of the ZIKV E protein are taken from the NCBI databank ( http://www.ncbi.nlm.nih.gov/nuccore/?term=zika) and are given in Dataset 1 (accessions: KU312314, KU312315, KU312313, KU312312, KJ776791, KJ634273, KF993678, JN860885, EU545988, HQ234499, KF268950, KF268948, KF268949, AY632535, LC002520, DQ859059, HQ234501, HQ234500, FSS13025, AHL43505, AHL43503, AHL43502, AMA12087). Sequences for HA1 from influenza viruses were taken from the GISAID database ( http://platform.gisaid.org) and are given in Dataset 2 (accessions: EPI705910, EPI696955).

Sequences of the ZIKV E protein taken from the NCBI databank (accessions: KU312314, KU312315, KU312313, KU312312, KJ776791, KJ634273, KF993678, JN860885, EU545988, HQ234499, KF268950, KF268948, KF268949, AY632535, LC002520, DQ859059, HQ234501, HQ234500, FSS13025, AHL43505, AHL43503, AHL43502, AMA12087) Click here for additional data file. Copyright: © 2016 Veljkovic V and Paessler S 2016 Data associated with the article are available under the terms of the Creative Commons Zero "No rights reserved" data waiver (CC0 1.0 Public domain dedication).

Sequences of HA of A/California/07/2009(H1N1) and HA of A/Switzerland/9715293/2013(H3N2) taken from the GSAID database (accessions: EPI705910, EPI696955) Click here for additional data file. Copyright: © 2016 Veljkovic V and Paessler S 2016 Data associated with the article are available under the terms of the Creative Commons Zero "No rights reserved" data waiver (CC0 1.0 Public domain dedication).

### Informational spectrum method

The ISM is a virtual spectroscopy technique, developed for the study of protein-protein interactions. The physical and mathematical base of ISM was described in detail elsewhere ( 5 and references therein), and here we will only in brief present this bioinformatics method.

The ISM technique is based on a model of the primary structure of a protein using a sequence of numbers, by assigning to each amino acid the correspondence value of the electron ion interaction potential (EIIP; L 0.0000, I 0.0000, N 0.0036, G 0.0050, V 0.0057, E 0.0058, P 0.0198, H 0.0242, K 0.0371, A 0.0373, Y 0.0516, W 0.0548, Q 0.0761, M 0.0823, S 0.0829, C 0.0829, T 0.0941, F 0.0946, R 0.0959, D 0.1263). The EIIP values are in Rydbergs (Ry).

The obtained numerical sequence is then subjected to a discrete Fourier transformation which is defined as follows:

$$\text{X}(\text{n})=\sum \text{x}(\text{m}){\text{e}}^{-\text{j}(2/\text{N})\text{nm}},\;\text{n}=1,2,\ldots ,\text{N}/2\;(1)$$

where x(m) is the m-th member of a given numerical series, N is the total number of points in this series, and X(n) are discrete Fourier transformation coefficients. These coefficients describe the amplitude, phase and frequency of sinusoids, which comprised the original signal. Relevant information encoded in the primary structure is presented in an energy density spectrum which is defined as follows:

$$\text{S}(\text{n})=\text{X}(\text{n}){\text{X}}^{*}(\text{n})={\left|\text{X}(\text{n})\right|}^{2},\;\text{n}=1,2,\ldots ,\text{N}/2.\;(2)$$

In this way, sequences are analyzed as discrete signals. It is assumed that their points are equidistant with the distance d = 1. The maximal frequency in a spectrum defined as above is F = 1/2d = 0.5. The frequency range is independent of the total number of points in the sequence. The total number of points in a sequence influences only the resolution of the spectrum. The resolution of the N-point sequence is 1/n. The n-th point in the spectral function corresponds to a frequency f(n) = nf = n/N. Thus, the initial information defined by the sequence of amino acids can now be presented in the form of the informational spectrum (IS), representing the series of frequencies and their amplitudes.

The IS frequencies correspond to the distribution of structural motifs with defined physicochemical properties determining a biological function of a protein. When comparing proteins, which share the same biological or biochemical function, the ISM technique allows detection of code/frequency pairs which are specific for their common biological properties, or which correlate with their specific interaction. These common informational characteristics of sequences are determined by the cross-spectrum (CS) which is obtained by the following equation:

C(j)=∏S(i,j)(3)

where Π(i,j) is the j-th element of the i-th power spectrum and C(j) is the j-th element of CS. Peak frequencies in CS represent the common information encoded in the primary structure of analyzed sequences. This information corresponds to the mutual interaction between analyzed proteins or their interaction with the common interactor.

## Results and discussion

**Figure 1.**
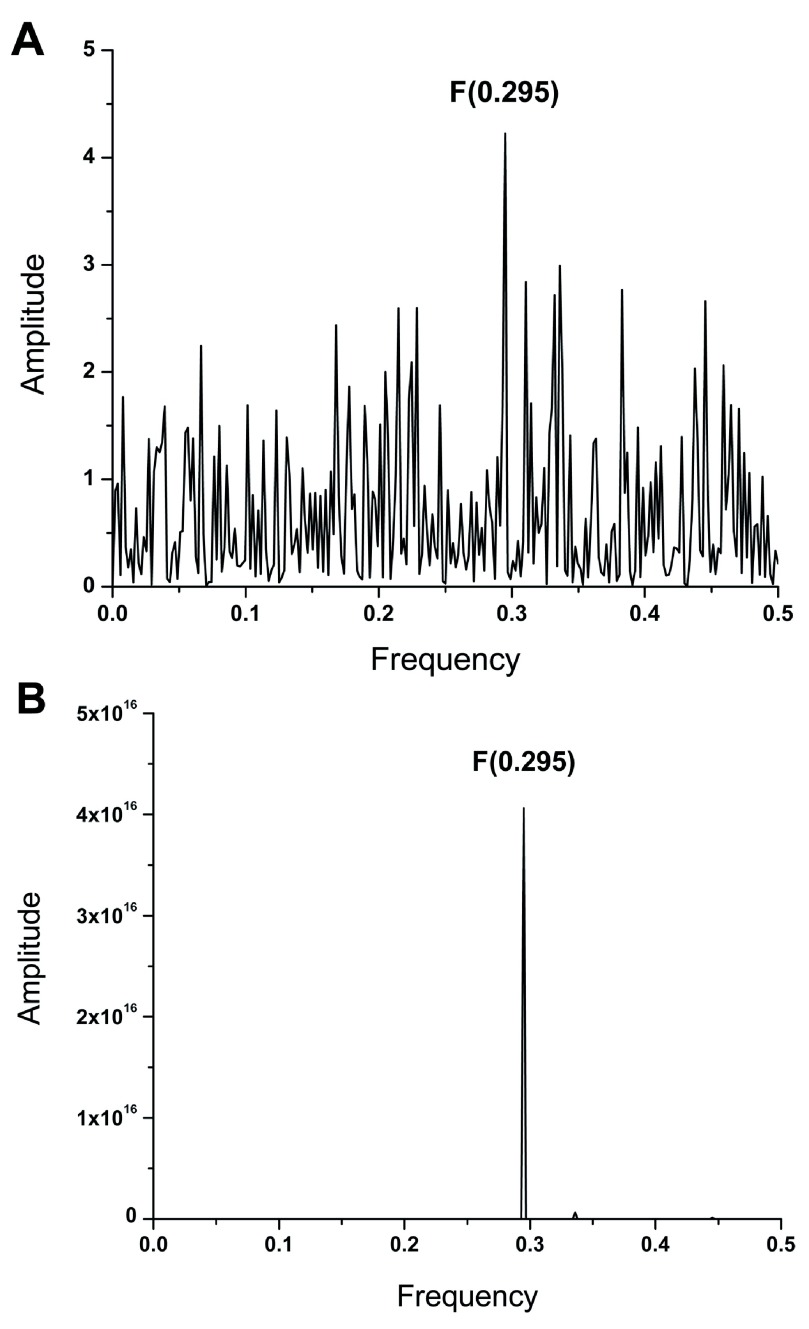
( **A**) The informational spectrum of ZIKV E protein. ( **B**) The consensus informational spectrum of protein E from ZIKV isolated in Asia, Africa and South/Central America.

The envelope glycoprotein (protein E) which mediates the virus cell entry is highly conserved and virtually identical in all ZIKV isolated during 2015 in countries of Central and South America. Figure 1A shows the IS of ZIKV E isolated in 2015 in Brazil (AMA12087) which is characterized by a dominant peak at frequency F(0.295). Figure 1B shows the CIS of protein E from ZIKV isolated between 1968 and 2015 in diverse countries of Asia and Africa ( Dataset 1). This CIS contains only one dominant peak at frequency F(0.295). This result suggests that all analyzed ZIKV E encode the same highly conserved information which is represented by the IS frequency F(0.295). According to the ISM concept ^6– 9^, this information determines the interacting profile of ZIKV E. 

**Figure 2.**
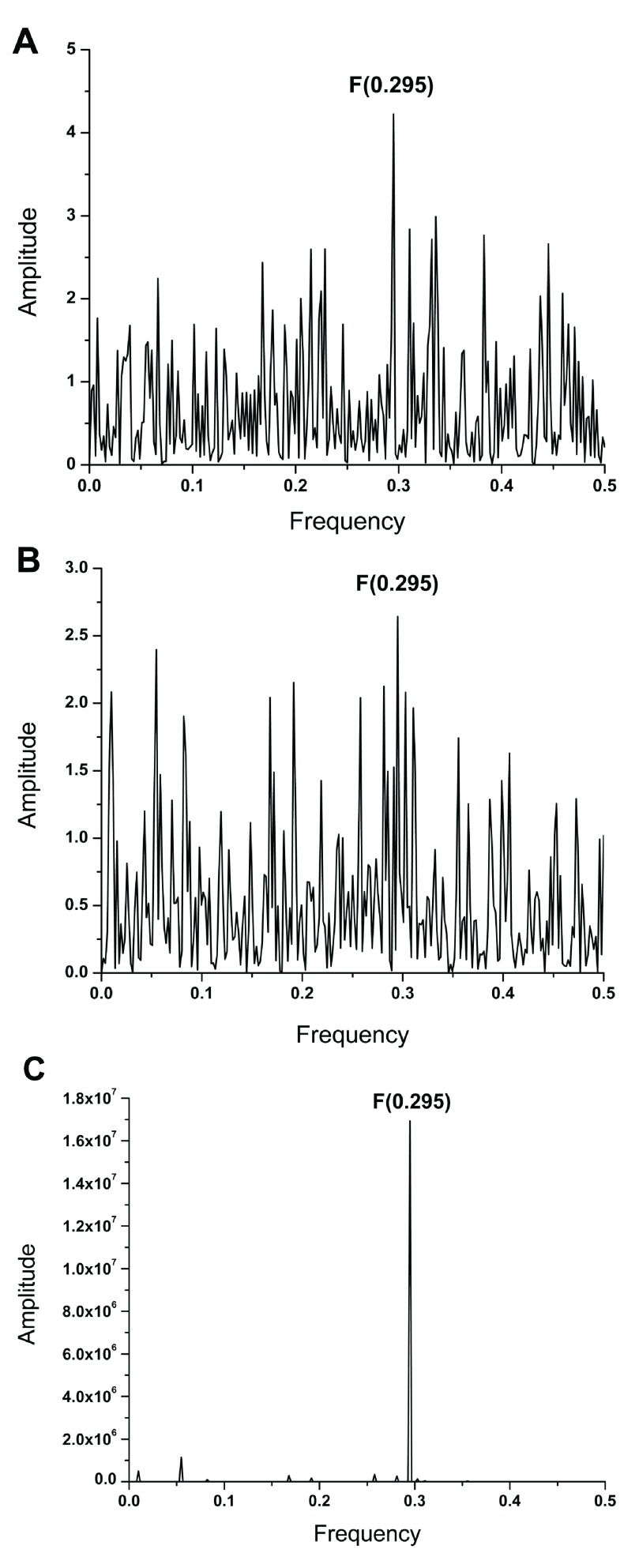
( **A**) The informational spectrum of ZIKV E protein. ( **B**) The informational spectrum of HA1 from A/California/07/2009(H1N1) influenza virus. ( **C**) The consensus informational spectrum of HA1 from pdmH1N1 ^2^.

**Figure 3.**
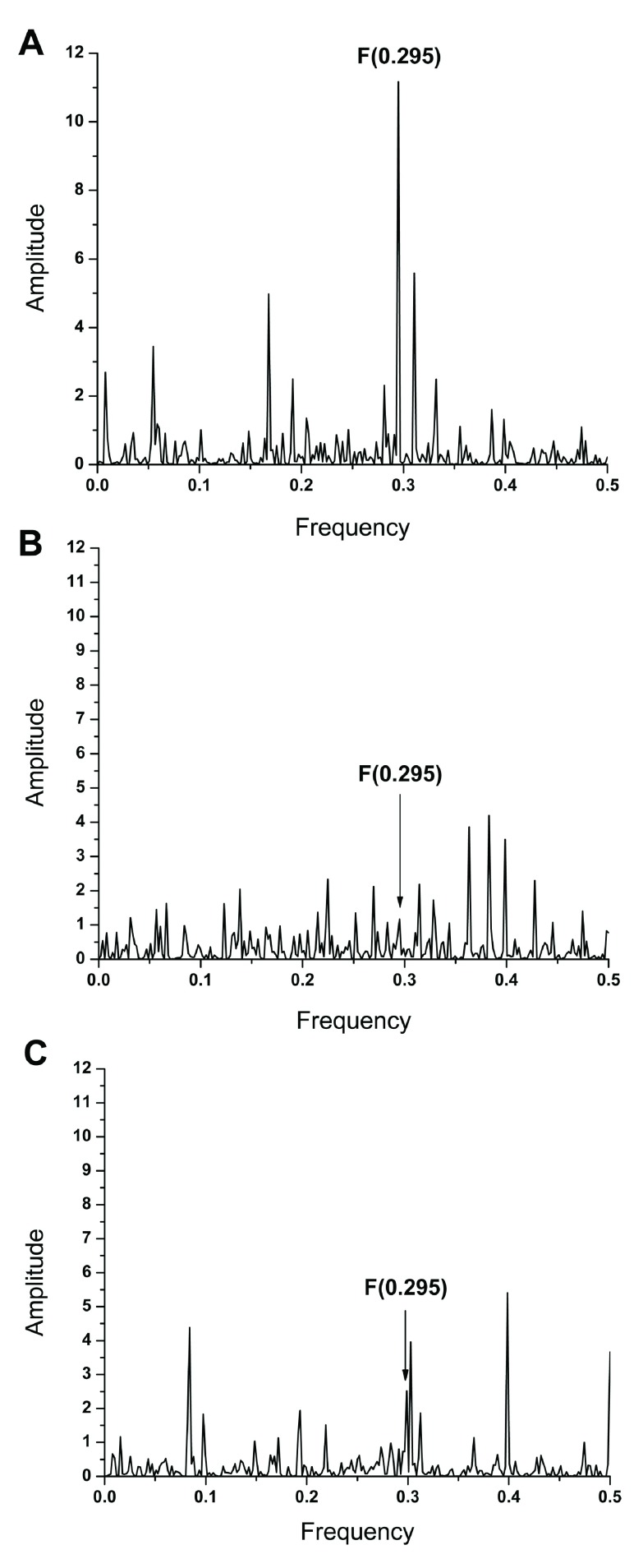
( **A**) The cross-spectrum of ZIKV E protein and HA1 from A/California/07/2009(H1N1) influenza virus. ( **B**) The cross-spectrum of ZIKV E protein and HA1 from A/Switzerland/9715293/2013(H3N2) influenza virus. ( **C**) The cross-spectrum of HA1 from A/California/07/2009(H1N1) and A/Switzerland/9715293/2013(H3N2) influenza viruses.

Previously, it has been shown that frequency F(0.295) characterizes the human interacting profile of pdmH1N1 HA1 ( Figure 2C) ^2^. It has also been shown that antigens which share a common frequency component in their IS are immunologically cross-reactive ( 10 and references therein). Presence of the frequency component F(0.295) in ZIKV E and pdmH1N1 HA indicates that antibodies elicited by this protein of influenza virus could affect interaction between ZIKV E and host proteins. We compared the IS of ZIKV E and HA1 from A/California/07/2009(H1N1) and A/Switzerland/9715293/2013(H3N2) viruses, which are components of the 2015/2016 seasonal influenza vaccine. Results given in Figure 2 and Figure 3A show that ZIKV E and A/California/07/2009(H1N1) encode the common information represented in their IS with the dominant peak at frequency F(0.295). As can be seen in Figure 3B & 3C, this frequency component is not present in the IS of A/Switzerland/9715293/2013(H3N2) HA1. These results indicate that antibodies elicited by the vaccine virus A/California/07/2009(H1N1) could affect interaction between ZIKA E and host proteins mediating virus cell entry.

## Conclusions

The presented results of the *in silico* analysis of ZIKV E and host factors mediating viral infection suggest that the seasonal influenza vaccines containing pdmH1N1 as a component, could protect to some extent against the ZIKV infection. Because of the lack of prevention and therapy of the ZIKV disease, in a situation when the ZIKV infection is explosively spreading, this possible safe and inexpensive solution is worth being seriously considered.

## Data availability

The data referenced by this article are under copyright with the following copyright statement: Copyright: © 2016 Veljkovic V and Paessler S

Data associated with the article are available under the terms of the Creative Commons Zero "No rights reserved" data waiver (CC0 1.0 Public domain dedication). 

*F1000Research*: Dataset 1. Sequences of the ZIKV E protein taken from the NCBI databank (accessions: KU312314, KU312315, KU312313, KU312312, KJ776791, KJ634273, KF993678, JN860885, EU545988, HQ234499, KF268950, KF268948, KF268949, AY632535, LC002520, DQ859059, HQ234501, HQ234500, FSS13025, AHL43505, AHL43503, AHL43502, AMA12087)., 10.5256/f1000research.8102.d114325 ^11^

*F1000Research*: Dataset 2. Sequences of HA of A/California/07/2009(H1N1) and HA of A/Switzerland/9715293/2013(H3N2) taken from the GSAID database (accessions: EPI705910, EPI696955)., 10.5256/f1000research.8102.d114326 ^12^
