# Supplementary material for: Possible repurposing of seasonal influenza vaccine for prevention of Zika virus infection
Source: F1000Res. 2016 Mar 23;5:190. Originally published 2016 Feb 18. [Version 2] doi: 10.12688/f1000research.8102.2 (PMC4857754; doi:10.12688/f1000research.8102.2)
Supplement: Peer review history

# Peer review: Referee response for version 2

**DOI:** 10.5256/f1000research.9037.r13043

**Published:** 2016-04-27

**Authors:** Patrizio Arrigo

**Affiliations:** 1 Institute for Macromolecular Studies, National Research Council, Genoa, Italy

I've checked the second revised version and it agrees with my suggestions. I guess it can now be indexed

I have read this submission. I believe that I have an appropriate level of expertise to confirm that it is of an acceptable scientific standard.

# Peer review: Referee response for version 1

**DOI:** 10.5256/f1000research.8717.r12533

**Published:** 2016-03-14

**Authors:** Heinz Kohler

**Affiliations:** 1 Department of Microbiology and Immunology, University of Kentucky, Lexington, KY, USA

This manuscript provides important information on the envelope of Zika virus that will help to produce a vaccine.

I have read this submission. I believe that I have an appropriate level of expertise to confirm that it is of an acceptable scientific standard.

# Peer review: Referee response for version 1

**DOI:** 10.5256/f1000research.8717.r12535

**Published:** 2016-03-03

**Authors:** Patrizio Arrigo

**Affiliations:** 1 Institute for Macromolecular Studies, National Research Council, Genoa, Italy

The paper summarizes an application of well established and efficient ISM method to compare ZIKA and H1N1. I would like to underline two points that I guess to be better describe. I suggest that Figure 2 can contain also the consensus spectrum as Figure 1.

The second point is the description of Figure 3 in the result section. I guess that the authors could extended the illustration of the differences of the cross-spectrum. These differences are very interesting data for discrimination. Another small remark is about the aim of the paper. The authors suggest a 'repositioning' of H1N1 vaccine if it is possible I would like to ask them if they have considered the possibility to elicit unfavourable effect of cross reactivity with the consequence to reduce the vaccine efficiency.

I have read this submission. I believe that I have an appropriate level of expertise to confirm that it is of an acceptable scientific standard, however I have significant reservations, as outlined above.
